# Exploration of the association of a lipid-related biomarker, the non-high-density lipoprotein cholesterol to high-density lipoprotein cholesterol ratio (NHHR), and the risk of breast cancer in American women aged 20 years and older

**DOI:** 10.1097/JS9.0000000000001700

**Published:** 2024-05-23

**Authors:** Xuemei Luo, Jianrui Ye, Ting Xiao, Tao Yi

**Affiliations:** a Department of General Surgery, Mianzhu City People's Hospital; b Department of Traditional Chinese Medicine, Longquanyi District Hospital of Traditional Chinese Medicine; c Department of Rehabilitation Medicine, Mianzhu City People’s Hospital, Mianzhu; d The School of Nursing, Chengdu Medical College, Chengdu, People’s Republic of China

*Dear Editor,*

The state of inflammation, metabolism, and other vital activities of the body have been shown to play a critical role in predicting the onset, recurrence, and prognosis of a wide range of diseases^1^. Recently, Xu *et al*.^1^ found that an elevated neutrophil-to-lymphocyte ratio (NLR) was associated with the development of stroke-associated pneumonia, and the functional outcome was worse with increasing NLR. This study reveals the potential of blood-related markers in predicting disease occurrence.

Breast cancer is the malignant tumor with the highest incidence rate among American women and also the main cause of cancer-related deaths among American women. It is reported that by 2024, it is estimated that there will be 310 720 new cases of breast cancer among American women, 42 250 deaths, and the incidence rate will increase year by year, reaching 0.6–1.0%^2^. The development and occurrence of breast cancer are the result of a variety of internal and external factors, including genetic factors, obesity, diabetes, the use of exogenous estrogen, and other factors^3,4^. The early clinical manifestation of breast cancer is not obvious and is easy to ignore, which makes breast cancer further progress and leads to poor prognosis for patients. Therefore, it is urgent to determine new biomarkers to assess individual susceptibility to breast cancer. At present, studies on dyslipidemia and the occurrence and development of breast cancer have been widely reported^5^. Non-high-density lipoprotein cholesterol to high-density lipoprotein cholesterol ratio (NHHR), as a blood-related marker of dyslipidemia, has been widely reported to be associated with and has good predictive value for the development of atherosclerosis, nonalcoholic fatty liver disease, chronic kidney disease, abdominal aortic aneurysm, diabetes mellitus, and depression, etc.^6^, however, the predictive role of NHHR in the development of breast cancer remains unknown. Therefore, this study analyzed data from the National Health and Nutrition Examination Survey (NHANES) 2009–2018 to investigate whether NHHR is associated with the risk of breast cancer in American women at least 20 years old.

According to the inclusion criteria and exclusion criteria, 9857 people were included in this study, including 293 people with breast cancer (Fig. 1). There are statistically significant differences between the breast cancer group and the nonbreast cancer group in terms of age, race, marital status, poverty impact ratio (PIR), hypertension, diabetes, whether ever pregnant, whether using estrogen drugs and total cholesterol. Unfortunately, no differences were found in NHHR (Table 1 in the supplementary material, Supplemental Digital Content 1, http://links.lww.com/JS9/C646). In order to avoid this result due to the influence of confounding factors, after adjusting the potential confounding factors, it is still not found that NHHR is associated with the risk of breast cancer (Table 2 in the supplementary material, Supplemental Digital Content 1, http://links.lww.com/JS9/C646). A total of 3971 participants were not included in this study due to missing data on covariates, independent variables, or outcome variables, which may have a certain impact on the results of this study, so the results of this study need to be treated with caution. In addition, prospective cohort studies should be used in the future to explore the correlation and causality between NHHR and the risk of breast cancer, with the aim of identifying high-risk groups for early intervention and improving the prognosis of breast cancer.

**Figure 1:**
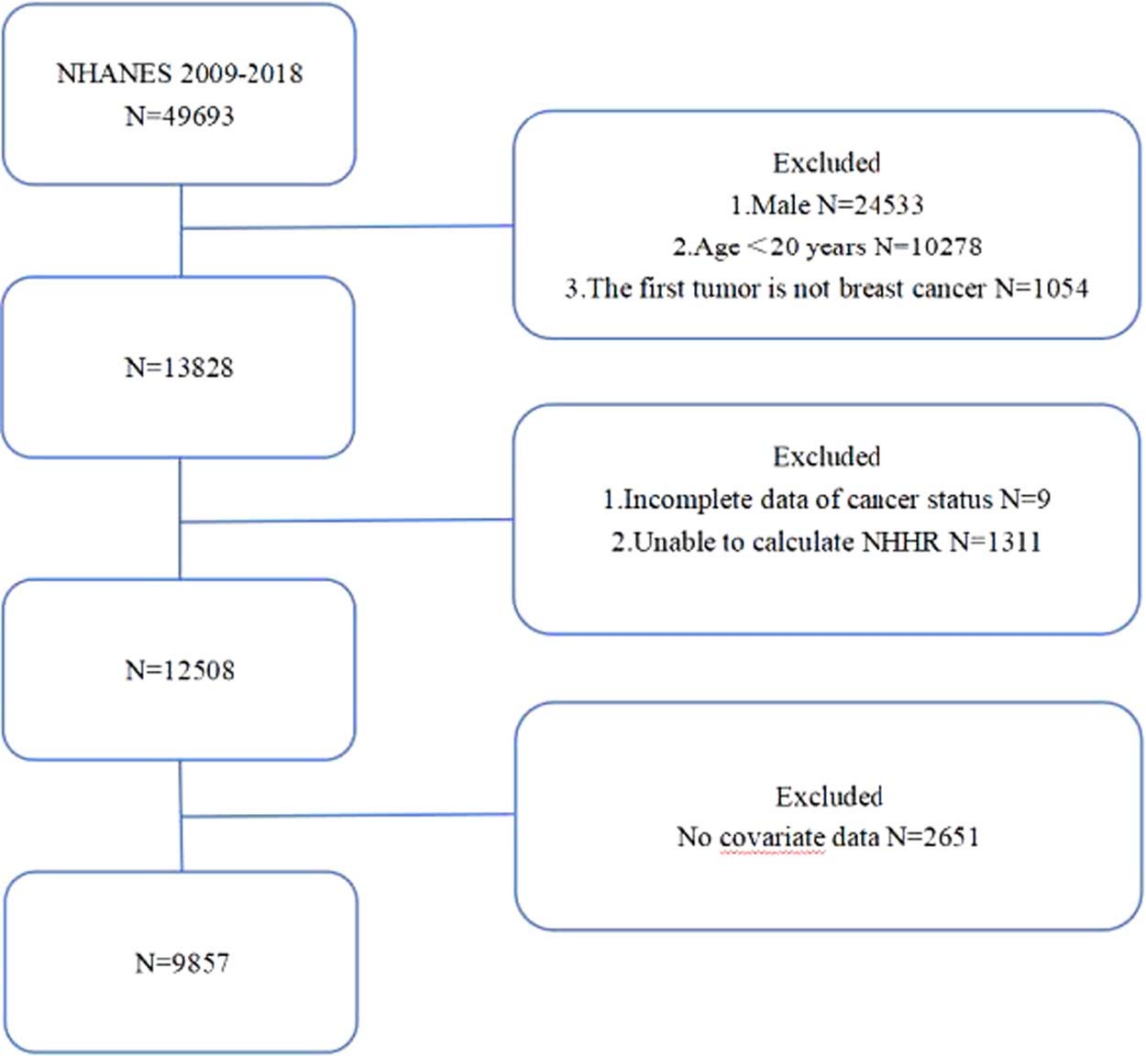
Flowchart of the participants’ selection from NHANES 2009–2018. NHANES, National Health and Nutrition Examination Survey; NHHR, non-high-density lipoprotein cholesterol to high-density lipoprotein cholesterol ratio.

## Ethical approval

The study used the NHANES database, which was endorsed by the NHANES Institutional Review Board, and all participants provided informed consent.

## Consent

The study used the NHANES database, which was endorsed by the NHANES Institutional Review Board, and all participants provided informed consent.

## Sources of funding

This work was supported by the Research Project Fund of the Science and Technology Bureau of Deyang City, Sichuan Province (No. 2023SZZ085).

## Author contribution

X.L.: study concept or design, data collection, data analysis or interpretation, and writing the paper; J.Y.: study concept or design, data collection, and writing the paper; T.X.: data collection; T.Y.: review paper.

## Conflicts of interest disclosure

The authors declare that there are no conflicts of interest regarding the publication of this paper.

## Research registration unique identifying number (UIN)

This is a retrospective study.

## Guarantor

Xuemei Luo and Jianrui Ye.

## Data availability statement

Publicly available datasets were analyzed in this study. This data can be found here: https://www.cdc.gov/nchs/nhanes/index.htm.

## Provenance and peer review

The paper was not invited.

## Supplementary Material
