# Supplementary material for: Exploration of the association of a lipid-related biomarker, the non-high-density lipoprotein cholesterol to high-density lipoprotein cholesterol ratio (NHHR), and the risk of breast cancer in American women aged 20 years and older
Source: Int J Surg. 2024 May 23;110(9):5939–41. doi: 10.1097/JS9.0000000000001700 (PMC11392120; doi:10.1097/JS9.0000000000001700)
Supplement: Supplementary file 1 [file js9-110-5939-s001.docx]

Table 1. Characteristics of participants

|  | Total(N=9857) | Breast cancer group(N=293) | Non-breast cancer group(N=9564) | P |
| --- | --- | --- | --- | --- |
| Age(years) | 48.00(34.00,62.00) | 69.00(60.00,77.00) | 47.00(33.00,62.00) | ＜0.001 |
| Race(%) |  |  |  | ＜0.001 |
| Mexican American | 1431 | 27 | 1404 |  |
| Other Hispanic | 1064 | 22 | 1042 |  |
| Non-Hispanic White | 3944 | 178 | 3766 |  |
| Non-Hispanic Black | 2126 | 42 | 2084 |  |
| Other Race | 1292 | 24 | 1268 |  |
| Education level(%) |  |  |  | 0.265 |
| Less than 9th grade | 810 | 22 | 788 |  |
| 9-11th grade (Includes 12th grade with no diploma) | 1214 | 33 | 1181 |  |
| High school graduate/GED or equivalent | 2111 | 62 | 2049 |  |
| Some college or AA degree | 3301 | 88 | 3213 |  |
| College graduate or above | 2421 | 88 | 2333 |  |
| Marital status(%) |  |  |  | ＜0.001 |
| Married | 4591 | 138 | 4453 |  |
| Widowed | 987 | 74 | 913 |  |
| Divorced | 1253 | 55 | 1198 |  |
| Separated | 373 | 7 | 366 |  |
| Never married | 1866 | 14 | 1852 |  |
| Living with partner | 787 | 5 | 782 |  |
| PIR(%) |  |  |  | ＜0.001 |
| 0-1.30 | 3335 | 68 | 3267 |  |
| 1.31-3.50 | 3693 | 118 | 3575 |  |
| 3.51-5.00 | 2829 | 107 | 2722 |  |
| BMI(kg/m2) | 28.65(24.23,34.10) | 28.46(24.73,34.53) | 28.67(24.20,34.10) | 0.464 |
| Hypertension(%) |  |  |  | ＜0.001 |
| Yes | 3506 | 180 | 3326 |  |
| No | 6351 | 113 | 6238 |  |
| Diabetes(%) |  |  |  | ＜0.001 |
| Yes | 1207 | 62 | 1145 |  |
| No | 8396 | 218 | 8178 |  |
| Borderline | 254 | 13 | 241 |  |
| Smoking status(%) |  |  |  | 0.395 |
| Yes | 3437 | 109 | 3328 |  |
| No | 3620 | 184 | 6236 |  |
| Age when first menstrual period occurred(years) | 13.00(12.00,14.00) | 13.00(12.00,14.00) | 13.00(12.00,14.00) | 0.524 |
| Ever been pregnant？(%) |  |  |  | ＜0.001 |
| Yes | 8204 | 269 | 7935 |  |
| No | 1653 | 24 | 1629 |  |
| Ever taken birth control pills？(%) |  |  |  | 0.083 |
| Yes | 6716 | 186 | 6530 |  |
| No | 3141 | 107 | 3034 |  |
| Ever use female hormones？(%) |  |  |  | ＜0.001 |
| Yes | 1644 | 86 | 1558 |  |
| No | 8213 | 207 | 8006 |  |
| Total Cholesterol (mg/dL) | 191.00(166.00,218.00) | 199.00(173.00,226.50) | 190.00(166.00,218.00) | 0.003 |
| HDL-C(mg/dL) | 55.00(46.00,67.00) | 57.00(47.00,67.50) | 55.00(46.00,67.00) | 0.149 |
| NHHR | 2.37(1.75,3.19) | 2.41(1.80,3.22) | 2.37(1.75,3.19) | 0.620 |

PIR, Poverty Impact Ratio; BMI, Body Mass Index; HDL-C, High Density Lipoprotein Cholesterol; NHHR, Non-high-density lipoprotein cholesterol to high-density lipoprotein cholesterol ratio.

| **Variables** | Model1 | | Model2 | | Model3 | |
| --- | --- | --- | --- | --- | --- | --- |
|  | OR (95%CI) | P | OR (95%CI) | P | OR (95%CI) | P |
| NHHR | 1.00 (0.91 ~ 1.10) | 0.986 | 0.98 (0.89 ~ 1.09) | 0.760 | 1.10 (0.80 ~ 1.51) | 0.549 |

Table 2. The association between NHHR and the risk of breast cancer in American women aged [20 years and older](https://pubmed.ncbi.nlm.nih.gov/37860196/)

NHHR: non-high-density lipoprotein cholesterol to high-density lipoprotein cholesterol ratio.

OR: odds ratio.

95% CI: 95% confidence interval.

Model 1: no covariates were adjusted.

Model 2: adjusted for age and race.

Model 3: adjusted for age, race, education level, marital status, poverty impact ratio, body mass index, hypertension, diabetes, smoking status, age when first menstrual period occurred, ever been pregnant？, ever taken birth control pills？, ever use female hormones？, total cholesterol and high density lipoprotein cholesterol.
